# Early pro-inflammatory host response to recombinant HSV-SIV vaccination in sooty mangabeys

**DOI:** 10.1186/1742-4690-9-S2-O17

**Published:** 2012-09-13

**Authors:** N Rout, S Yu, V Varner, C Kasala-Hallinan, K Rogers, J Sen, D Knipe, F Villinger, A Kaur

**Affiliations:** 1 NEPRC-HMS, Southborough, MA, USA; 2 YNPRC-Emory University, Atlanta, GA, USA; 3 Harvard Medical School, Boston, MA, USA

## Background

Naturally infected sooty mangabeys (SM) do not progress to AIDS unlike SIV-infected non-natural hosts including rhesus macaques (RM). Differences in the innate immune response to SIV may be critical in avoidance of disease progression in SIV-infected SM. In this study we investigated innate and adaptive immune responses to recombinant HSV vectors expressing SIV Gag, Env, and a Rev-Tat-Nef fusion protein in six SIV-negative SM and four RM following single intramuscular inoculation.

## Methods

Plasma samples were analyzed using Luminex 28-plex assay. Innate immune cells and SIV-specific cellular immune responses were investigated by flow cytometry and IFN-γ ELISPOT assay.

## Results

A significant transient decline of circulating Natural Killer T (NKT) cells, pDCs, and NK cells was observed in SM on day one post vaccination, with a return to baseline levels by day 3-7. Also an increased activation of SM NK cells was observed that returned to baseline by day 9. In contrast, RM displayed no significant change in circulating NKT cell and NK cell frequencies. Both RM and SM exhibited similar transient increases in plasma levels of several inflammatory cytokines/chemokines including IL-1RA, IFN-γ, IL-6, IL-8, macrophage migration inhibitory factor (MIF), monocyte chemotactic protein 1 (MCP-1), vascular endothelial growth factor (VEGF), and interferon-inducible T cell alpha-chemoattractant (I-TAC) by day one, suggesting activation of multiple immune cells including macrophages and DCs. By day 14, plasma levels of analytes such as IL-6 and Eotaxin persisted at elevated levels in RM, but not SM, suggesting delayed resolution of immune activation in RM. SIV-specific cellular immune responses were detected in both species.

## Conclusion

These data confirm that SM mount a robust innate immune response and do not have blunted immune activation. Further investigation of the mechanism by which SM resolve the transient but higher immune activation would be helpful in improving vaccine strategy.

